# Selective arterial embolization as an alternative modality treatment prior to mandibular aneursymal bone cyst surgical curettage: Case report

**DOI:** 10.4317/jced.59530

**Published:** 2022-12-01

**Authors:** Conrado Andrés-Ros, Eduardo Pérez-Fernández, Beatriz García-Carballo, Soledad Fernández-Solé, Leyre Margallo-Itza, Estibaliz Ortiz-de Zárate-Román

**Affiliations:** 1 MD Resident, Oral and Maxillofacial Surgery Department, Cruces University Hospital. Barakaldo, Spain; 2 MD, Pathology Department Cruces University Hospital. Barakaldo, Spain; 3 MD, Oral and Maxillofacial Surgery Department, Cruces University Hospital. Barakaldo, Spain; 4 MD, PhD. Head of Oral and Maxillofacial Surgery Department, Cruces University Hospital. Barakaldo, Spain. University Professor in the Department of Stomatology of the Faculty of Medicine and Dentistry of the UPV/EHU (University of the Basque Country). Professor at the School of Master and Doctorate of the UPV/EHU

## Abstract

ABC (Aneurysmal bone cyst) is a rare, benign and osteolytic lesion. Diagnosis of ABC can be challenging because of its uncommon radiographic and clinical presentation. The case of an 8-year-old female with a rapidly growing painful swelling in her left mandible is presented. Incisional biopsy showed an aneurysmal bone cyst. Surgical curettage and en bloc surgical excision are the main choices of treatment. We report embolization technique as a complement treatment for surgical curettage with accurate zone control lessening the extent of operation and surgical complications.

** Key words:**Aneurysmal bone cyst, embolization, mandible.

## Introduction

ABC is defined as a non-neoplastic lesion of the bone characterized by replacement with fibro-osseous tissue containing blood-filled sinusoidal spaces (1). The term “aneurysmal” describes its radiographic ballooned appearence of cortex. Its ethiology it is still unclear. It occurs in the second decade of age with female preponderance (2-5). Represents 1% of all primary bone tumours (4-6). Location is more common in long bones and spine and rare in jaws, only 1.9% of all ABCs (2). It is important to distinguish this anomaly from other mandible pathological entities, so we do not misdiagnosed it: Ameloblastoma, telangiectatic osteosarcoma, myxofibroma, giant cell granuloma, acute osteomyielitis or odontogenic cysts (1-6). Surgical curettage, segmental resection, radiotherapy, sclerosing injections and embolization are varieties of treatment described. We emphazise its clinic course, diagnosis and embolization as an alternative modality treatment prior to its surgical excision.

## Case Report

**Figure 1:**
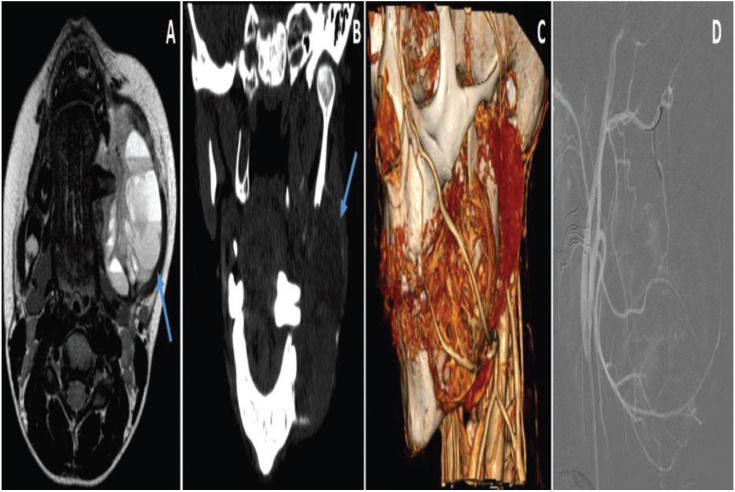
A: Axial T2 MRI (Magnetic Resonance Imaging) shows fluid-fluid levels characteristic of ABC (Arrow). B: CT (Computerized tomography) coronal view, shows a corticated (Arrow) ballon-like expansile lesion in the left posterior mandible with tooth desplazement. C: Lateral view of CT Angiography volume rendering. D: Angiography showing embolization procedure.

**Figure 2:**
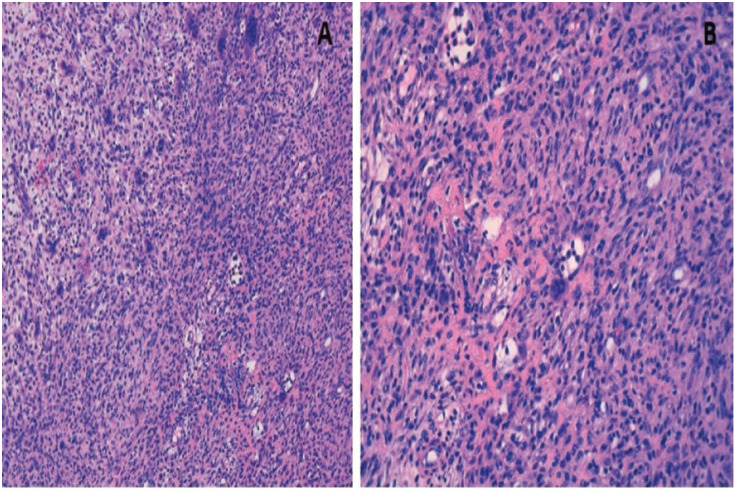
Proliferation of spindle cells is observed, in a stroma with predominantly loose, edematous areas, with a prominent vascular pattern and frequent giant cells of the osteoblast type. There is presence of osteoid matrix. A: Hematoxylin & Eosin stain(HE) (10x). B: HE (20x).

**Figure 3:**
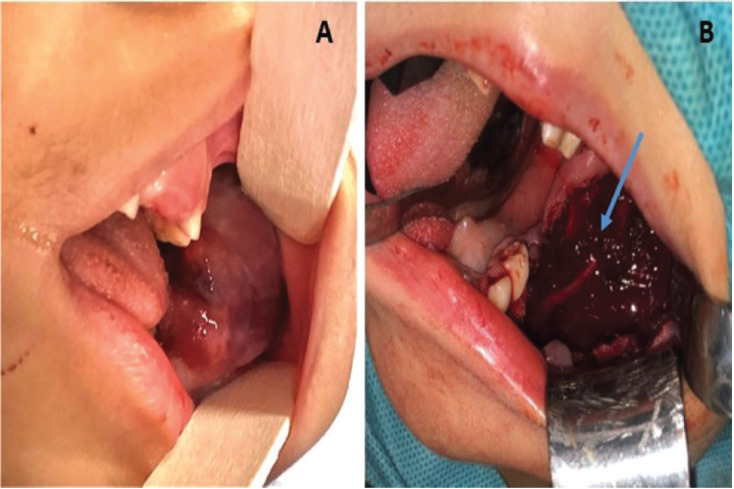
A: Intraoral mass in the area of the body and angle of the left mandible. B: Curettage vía intraoral approach preserving IAN (Arrow).

A case of ABC in an 8-year-old female is presented. Consults for a two month-old rapidly growing painful swelling of the left mandible. No significant medical history, apart from mother’s Chagas disease nor history of trauma. Physical examination shows an intraoral mass in body and angle of the left mandible of about 5cm causing regional tooth displacement (Figs. 1-3). No paresthesia. No palpable lymphadenopaties are identified. Normal values in hematologic studies. Orthopantomography and a CT (Computerized Tomography) scan are firstly done showing “ballooning” expansion (Figure 1B,C). Magnetic Resonance Imaging (MRI) is done to complete imaging diagnosis showing loculations and fluid-fluid levels (2,4,6-8) (Fig. 1A). Intraoral incisional biopsy and FISH (Fluorescence in situ hybridization) was performed to detect USP6 gene rearrangements detectable in 70% of primary ABC (9). Based on histology (Fig. 2) and imaging studies, a provisional diagnosis of an ABC is made.

As this ABC was classified as a vascular type lesion, prior to definitive curettage of the lesion, a vascular map of the feeding vessels was obtained and supraselective catheterizations with the following embolization with Embosphere® 300-500 microns (Tris-acryl microspheres) is performed (Fig. 1D). Embolization of branches from inferior alveolar artery, posterior superior alveolar artery and facial artery are done. 24 hours later under general anesthesia surgical curettage via intraoral approach without bone grafting is performed. The inferior alveolar nerve (IAN) is preserved. After 3 month follow-up no recurrence is present.

## Discussion

The term ABC was introduced by Jaffe and Lichtenstein in 1942(10). Bernier and Bhaskar reported the first case involving the mandible in 19588. The term “aneurysmal bone cyst” is confusing. It is not “aneurysmal” because there is no endotelial lining of the vascular spaces, and it is classified as “pseudocyst” as it is a cyst-like structure without an epithelial lining (3,7). The ethiology remains unknown and it is still controversial. Several authors (8-10) defend ABC pathogenesis is due to a local proliferative vascular response to unknown stimulus in the form of a sudden venous occlusion with dilatation of the vascular network or the development of an arterio-venous shunt in between the lesion stroma and the medullary vessels (5-8). A predisposing genetic defect could be part of a multifactorial pathogenesis in the development of some ABCs. Primary ABCs are a mesenchymal neoplastic disease characterized by a spindle cell proliferation exhibiting USP6 or CDH11 rearrangements in chromosomal region 17p13.2. In other entitities such as giant cell tumor, osteosarcoma, osteoblastoma, brown tumor, vascular neoplasms this rearrangement is not present (6,9).

Craniofacial skeleton is a rare presentation site however the mandible, particularly retromolar area, as in the case presented (Fig. 3), is more affected than the maxilla (mandible-maxilla ratio, 3:1) (1). Other facial skeletal areas have been described such as the infratemporal region, zygoma and orbital floor. The mean age of presentation is 14.3 years. Asymptomatic at onset but they usually have a rapid growth causing expansion and destruction of the surrounding bone structures, sometimes imitating a malignant tumor (1,5,6,8). This leads to its main symptons when established: pain and/or edema. Other associated symptons depending on which area is affected can also be present: Paresthesia, migration or resorption of involved teeth, proptosis, epistaxis, nasal obstruction…

Radiographic characteristics are not pathognomonic and definite radiographic diagnostic criteria should be established for future studies. The lesion morphology varies and can become very large (>10cm) (2). They may appear unicystic showing less expansion, multilocular with balloning expasion of mandibular cortex or moth eaten (1,3). However fluid-fluid levels seen in MRI representing the blood-filled sinusoids is the best diagnostic clue (Fig. 1). It clasiffies in solid (5% of cases), vascular and mixed type. Solid type is smaller, often asymptomatic with less swelling and bleeding while vascular type (this is the one we are introducing) is a more expansile and destructive lesion with excessive bleeding at surgery. The third, mixed type: Possibly represents a transition phase between the other two.

The gold standard treatment for ABCs is open surgical curettage of the lesion. Nevertheless, therapeutic options will depend on the size, position, patient age and the pathology’s extension. In adults and in very bulky lesions to perform subperiostal resection it is not technically viable. It is posible in children like in the case we are analyzing. The remaining periosteum will contribute to good bone consolidaton. Other treatments are described such as: sclerotherapy, en bloc resection or primary therapeutic embolization. Radiotherapy is not recommended because of the probability of radiation-induced sarcoma and its high failure rate (1,3,7).

Massive bleeding is the main complication throughout surgical curettage especially in vascular type lesions, like the one we are studying. Selective arterial embolization is indicated in the treatment of ABC, especially in these patients at high risk of extensive intraoperative bleeding.

Embolization with resorbable materials and/or coils, was initially introduced as a preoperative procedure to reduce intraoperative hemorrhage and for the treatment of sites with difficult surgical access. Percutaneous intralesional injection with fibrosing agents, such as Alcoholic zein (Ethibloc) (4,5,7) was also used as an alternative to surgery. Kumar *et al*. (7) employed transcutaneous intralesional embolization with diluted n-butyl-cyano acrylate glue as an adjunct to resection.

Benefits from embolization in the reported case include: a) decrease blood supply to limit intraoperative blood loss and the need for multiple blood transfusions; b) achieve bloodless surgical field which allows complete removal of the lesion reducing recurrence c) decrease surgical time. Recurrence rate after curettage in the jaws vary widely in literature, from 0 to 53%(8) No recurrence was reported in this case however a long-term follow-up is required. Some authors defend that selective arterial embolization should be considered as the treatment of choice for almost all ABCs (5).

Massive hemorraghe in need of ligation of the external carotid artery has also been described in order to diminish bleeding risk (7). In our experience, embolization prior to surgical curettage was associated with lower complication rates. Postoperative elapses without incidents. Histopathological definitive diagnosis shows fibrous septa with giant cells and lined with flattened fibroblasts and blood-filled caverns (Fig. 2).

Articles from the literature where evaluated showing different forms of embolization of ABC localized in the appendicular skeleton (4,5). To our knowledge there are few studies in literature regarding the use of preoperative SAE (Selective arterial embolization) for mandibular lesions (6).

In conclusion, the present case shows a primary ABC affecting a child where aggresive surgery has been avoided by the use of embolization prior to mandibular surgical curettage. Either percutaneous or selective arterial lesion embolization, seem a useful procedure to improve surgical outcomes as it avoids intra-operative bleeding.
